# The regulation of *ZCT1*, a transcriptional repressor of monoterpenoid indole alkaloid biosynthetic genes in *Catharanthus roseus*

**DOI:** 10.1002/pld3.193

**Published:** 2019-12-31

**Authors:** Samuel Mortensen, Jessica D. Weaver, Suphinya Sathitloetsakun, Lauren F. Cole, Noreen F. Rizvi, Erin J. Cram, Carolyn W. T. Lee‐Parsons

**Affiliations:** ^1^ Department of Biology Northeastern University Boston MA USA; ^2^ Department of Chemistry and Chemical Biology Northeastern University Boston MA USA; ^3^ Department of Bioengineering Northeastern University Boston MA USA; ^4^ Department of Chemical Engineering Northeastern University Boston MA USA

**Keywords:** *activation sequence‐1*‐like, *Agrobacterium*‐mediated transformation, *Catharanthus roseus*, Cys_2_/His_2_‐type zinc finger transcription factors, ORCA, plant stress response

## Abstract

Cys_2_/His_2_‐type (C_2_H_2_) zinc finger proteins, such as ZCT1, are an important class of transcription factors involved in growth, development, and stress responses in plants. In the medicinal plant *Catharanthus roseus,* the zinc finger *Catharanthus* transcription factor (ZCT) family represses monoterpenoid indole alkaloid (MIA) biosynthetic gene expression*.* Here, we report the analysis of the *ZCT1* promoter, which contains several hormone‐responsive elements. *ZCT1* is responsive to not only jasmonate, as was previously known, but is also induced by the synthetic auxin, 1‐naphthalene acetic acid (1‐NAA). Through promoter deletion analysis, we show that an *activation sequence‐1*‐like (*as‐1*‐like)‐motif and other motifs contribute significantly to *ZCT1* expression in seedlings. We also show that the activator ORCA3 does not transactivate the expression of *ZCT1* in seedlings, but ZCT1 represses its own promoter, suggesting a feedback mechanism by which the expression of *ZCT1* can be limited.

## 1 INTRODUCTION

Due to their sessile existence, plants have developed rapid and effective responses to environmental stresses, herbivory, and pathogens. When stressed, plants can shift primary metabolic resources to produce specialized metabolites that act as defense compounds. For instance, the Madagascar periwinkle, *Catharanthus roseus*, produces numerous monoterpenoid indole alkaloids (MIA) in defense against pathogens and herbivory (Luijendijk, van der Meijden, & Verpoorte, 1996; Dugé de Bernonville et al., 2017). Many of these MIAs are pharmaceutically valuable, including the antihypertensive ajmalicine, the sedative serpentine, and most notably the anticancer compounds vincristine and vinblastine (Van der Heijden, Jacobs, Snoeijer, Hallard, & Verpoorte, 2004; Noble, 1990).

A crucial regulatory mechanism in specialized metabolism is the transcriptional regulation of biosynthetic genes. Several transcription factors (TFs) involved in MIA regulation have been identified in *Catharanthus*, including the octadecanoid‐responsive *Catharanthus* AP2‐domain (ORCA) TF family and the zinc finger *Catharanthus* (ZCT) TF family described below (Menke et al., 1999; Van der Fits & Memelink, 2000; Van der Fits, Zhang, Menke, Deneka, & Memelink, 2000; Sibéril et al., 2001; Chatel et al., 2003; Pauw et al., 2004; Vom Endt, Soares e Silva, Kijne, Pasquali, & Memelink, 2007; Zhang et al., 2011; Suttipanta et al., 2011; Van Moerkercke et al., 2015, 2016; Paul et al., 2016; Liu et al., 2017; Liu, Patra, Pattanaik, Wang, & Yuan, 2019; Patra, Pattanaik, Schluttenhofer, & Yuan, 2017; Pan et al., 2019). Three characterized members (*ZCT1*, *ZCT2,* and *ZCT3*) of the Cys_2_/His_2_‐type (C_2_H_2_) zinc finger family are expressed by the stress‐induced phytohormone jasmonate (Goklany, Rizvi, Loring, Cram, & Lee‐Parsons, 2013; Pauw et al., 2004). ZCTs repress the expression of at least two of the key MIA biosynthetic genes, strictosidine synthase (*STR*) and tryptophan decarboxylase (*TDC*), in transient expression assays (Mortensen et al., 2019; Pauw et al., 2004).

ZCTs potentially limit the extent of MIA biosynthesis induced by jasmonate. For instance, optimum dosages of jasmonate (up to 250 μM; Goklany et al., 2013; Lee‐Parsons, Ertürk, & Tengtrakool, 2004) enhance MIA biosynthesis and are correlated with a high ratio of transcriptional activators (*ORCAs*) to repressors (*ZCTs*) levels (Goklany et al., 2013). But higher dosages of jasmonate (>500 μM; Goklany et al., 2013; Lee‐Parsons et al., 2004) inhibit MIA biosynthesis and are correlated with a high ratio of transcriptional repressors (*ZCTs*) to activator (*ORCAs*) levels (Goklany et al., 2013). The inhibition of MIA biosynthesis with high jasmonate dosages is potentially mediated through repressors like ZCTs. The repressor activity of the ZCTs is conferred by an ERF‐associated amphiphilic repression (EAR)‐motif (Ohta, Matsui, Hiratsu, Shinshi, & Ohme‐Takagi, 2001). A detailed description of the *C. roseus* ZCTs can be found in Pauw et al., 2004, and a more general characterization of zinc finger proteins is given in Kiełbowicz‐Matuk, 2012.

*Catharanthus roseus* ZCTs belong to the C_2_H_2_‐type zinc finger family. Analysis of the *Arabidopsis thaliana* genome revealed that C_2_H_2_‐type zinc fingers represent a large family of TFs. Of the 176 C_2_H_2_‐type zinc fingers identified in *A. thaliana*, only 33 are conserved among other eukaryotes, and the rest are plant‐specific; these plant‐specific C_2_H_2_‐type zinc fingers result from extensive duplication events (Englbrecht, Schoof, & Böhm, 2004). Conserved C_2_H_2_‐type zinc fingers are believed to be involved in ancient biological processes such as RNA metabolism and chromatin remodeling, whereas the duplicated zinc fingers may be involved in species‐specific transcriptional regulation (Englbrecht et al., 2004). In plants, C_2_H_2_‐type zinc fingers are involved in stress responses, for example, cold, drought, salt, and oxidative stress responses (Ciftci‐Yilmaz & Mittler, 2008; Kiełbowicz‐Matuk, 2012). The *C. roseus* zinc finger proteins ZCT1, ZCT2, and ZCT3 have the highest similarity, based on a blastp search, to ZAT8 (AT3G46080), ZAT17 (AT2G28710), and ZAT10/STZ (AT1G27730), respectively, in the *A. thaliana* genome. All three *A. thaliana* zinc finger proteins are C1 family and C1‐2i subclass members (Englbrecht et al., 2004). *ZAT10* is one of the better studied TFs of the C1‐2i subclass members and is responsive to a number of abiotic stressors such as abscisic acid (ABA), cold temperatures, high light intensities, oxidative stress, salt stress, water deprivation, and wounding (Mittler et al., 2006; Sakamoto, Araki, Meshi, & Iwabuchi, 2000; Sakamoto et al., 2004).

Similarly, *C. roseus ZCT1‐3* expression has been shown to be responsive to stressors and is induced by yeast extract, methyl jasmonate (MJ) (Goklany et al., 2013; Pauw et al., 2004), and sodium nitroprusside (SNP), a source of the signaling molecule nitric oxide (Zhou, Zhu, Shao, Wu, & Tang, 2010). Overexpression of *ORCA3*, one of the key MIA pathway activators, correlates with increased *ZCT* expression in *C. roseus* hairy roots, suggesting a potential regulation of *ZCTs* by ORCA3 (Peebles, Hughes, Shanks, & San, 2009). Otherwise, very little is known about the regulation of *ZCTs.*

Here, we have chosen to investigate the 1000‐bp upstream region of the *ZCT1* coding sequence as the *ZCT1* promoter contains many putative hormone‐responsive elements, and *ZCT1* expression is highly induced upon MJ treatment in hairy roots (Goklany et al., 2013). Based on these putative regulatory elements and the role of C_2_H_2_‐type zinc fingers, we explored whether endogenous *ZCT1* expression responds to hormones associated with growth, biotic, and abiotic stress such as auxin, gibberellin (GA), methyl jasmonate (MJ), salicylic acid (SA), and abscisic acid (ABA). We report that auxin (1‐NAA) and MJ induce *ZCT1* expression in hairy roots. Through promoter analysis experiments in transiently transformed seedlings, we determined that an *activation sequence‐1*‐like motif (*as‐1*‐like motif) contributes significantly to the promoter activity of *ZCT1*. Further elements within the promoter, including a GA‐responsive complex (GARC), also contribute. Overexpression of ORCA3 did not transactivate the expression of *ZCT1* or activate *ZCT1* promoter–reporter constructs in seedlings. ZCT1 is capable of repressing its own promoter, suggesting a possible feedback mechanism limiting the expression of *ZCT1*.

## 2 MATERIALS AND METHODS

### 2.1 Cloning, sequencing, and in silico analysis of the *ZCT1 *promoter

The *ZCT1* promoter sequence was obtained with the Universal Genome Walker Kit (Clontech). Approximately 800 bp of the *ZCT1* promoter was amplified from *C. roseus* (Little Bright Eye, NEseeds) genomic DNA using the GSP1 and GSP2 gene‐specific primers (Table S1) matching the *ZCT1* coding sequence (GenBank accession AJ632082). This sequence matches the sequence 5′ of the *ZCT1* coding region of the published genomes (Franke et al., 2019; Kellner et al., 2015). The promoters of *C. roseus ZCT2* and *ZCT3* were obtained from the published genomes (Figure S1 and Supplemental Materials).

To identify the transcriptional start site, the SMARTer RACE cDNA Amplification Kit (Clontech) was used to amplify the 5′ cDNA ends using the same GSP1 and GSP2 gene‐specific primers. Promoter sequences were analyzed with the Plant Cis‐Acting Regulatory Elements (PlantCARE; Lescot et al., 2002) and PlantPAN 3.0 databases (Chow et al., 2019). Using the *A. thaliana* PlantPAN 3.0 database, only exactly matching motifs (similar score of 1) were further considered. Motifs with low information content (e.g., GAT, TF_motif_seq_0237) and frequent occurrences in all tested promoters were excluded. The pyrimidine box matches sequences from the literature (Skriver, Olsen, Rogers, & Mundy, 1991; Rogers, Lanahan, & Rogers, 1994; Gubler et al., 1999).

### 2.2 Creation of a sequence logo for* as‐1*‐like sequences and *C. roseus ZCT1 as‐1*‐like sequence

*As‐1*‐like elements from *A. thaliana GST6* (Chen, Chao, & Singh, 1996), *A. thaliana PR‐1* (Zhang, Fan, Kinkema, Li, & Dong, 1999), *Agrobacterium tumefaciens MAS* (Feltkamp, Masterson, Starke, & Rosahl, 1994), *A. tumefaciens NOS* (Bouchez, Tokuhisa, Llewellyn, Dennis, & Ellis, 1989), *A. tumefaciens OCS* (Bouchez et al., 1989), *Cauliflower mosaic virus 35S* (Liu & Lam, 1994), *Glycine max GH2/4* (Ulmasov, Hagen, & Guilfoyle, 1994), *Nicotiana tabacum GNT1* (van der Zaal et al., 1991), *N. tabacum GNT35* (van der Zaal et al., 1991), *N. tabacum 103* (Droog, Hooykaas, Libbenga, & Zaal, 1993), *N. tabacum PR‐1a* (Strompen, Grüner, & Pfitzner, 1998), *N. tabacum ParA* (Takahashi, Kusaba, Hiraoka, & Nagata, 1991), *Silene cucubalis GST* (Prändl & Kutchan, 1992)*,* and *Triticum aestivum GST‐A1* (Dudler et al., 1991 *;* reviewed in Ulmasov et al., 1994 and Krawczyk et al., 2002) were used to create a WebLogo (Crooks et al., 2004). The spacer region was excluded from the WebLogo.

### 2.3 Cloning* ZCT1* promoter constructs for transient evaluation and stable infections

For vector construction, the Golden Gate‐based modular cloning system (MoClo, Weber, Engler, Gruetzner, Werner, & Marillonnet, 2011) was used, with parts from Engler et al. (2014) and Mortensen et al. (2019).

A 1000‐bp (−914 bp to +86 bp, with TSS set to +1) fragment of the *ZCT1* promoter was amplified from *C. roseus* (Little Bright Eye) genomic DNA and cloned into pICH41295 (Weber et al., 2011). The five promoter deletions were generated using standard cloning techniques. The mutant *as‐1*‐like element is identical to the *pZCT1_744* promoter construct, except for the reverse complementary *as‐1*‐like sites (first and last 5 bp were replaced with reverse complementary sequence) and was purchased from Genewiz in pUC57. The 35S minimal promoter (−46‐ to +6‐bp region) was amplified from pICH51288 (Engler et al., 2014) and cloned into pICH41246 (5U/5U + NT1) and pICH41295 (Pro + 5U; Weber et al., 2011). This allows the fusion of different regulatory elements to the 35S minimal promoter (in pICH41246) for studying its promoter activity isolated from the rest of the *ZCT1* promoter. The 35S minimal promoter alone (in pICH41295) serves as a reference. The GA‐responsive complex (GARC; 142 bp) and the GARC with the second INDETERMINATE DOMAIN (IDD) binding site (173 bp) were cloned into pICH41233 (Weber et al., 2011). Promoter elements and deletions were then assembled to a firefly luciferase reporter gene in level 1 vectors as described in Weber et al., (2011). The firefly luciferase (*FLUC*) reporters were cloned together with a constitutively expressed *Renilla* luciferase (*RLUC*) for normalization (Mortensen et al., 2019). The final constructs were assembled in the pSB90 (Mortensen et al., 2019) vector backbone. The pSB90 vector contains *VirGN54D* in the vector backbone for increased *Agrobacterium* virulence (as demonstrated in Mortensen et al., (2019)). All newly cloned parts were confirmed by sequencing, and correct assembly was confirmed by restriction enzyme digest, PCR, and/or sequencing. Vector cartoons can be found in Figure S4, and promoter sequences are provided as Supplemental Materials.

### 2.4 Preparation of *C. roseus* seedlings for developing transgenic hairy root lines

*Catharanthus roseus* seeds (Little Bright Eye) were surface‐sterilized in 70% (v/v) ethanol for one minute, followed by 10% (v/v) bleach containing 0.1% (v/v) Triton X‐100 for ten minutes. The seeds were rinsed three times in sterile water and soaked in 1% Plant Preservative Mixture (Caisson Laboratories) in sterile water for 24 hr. The seeds were planted in sterile Magenta GA‐7 boxes on the surface of 1/2 strength Murashige and Skoog media (2.2 g/L Murashige and Skoog basal salts with vitamins, 3% sucrose, 4 g/L Phytoagar, pH 5.7). Seedlings were grown in the dark at 25°C for one week and then transferred to a 16 hr of light (Erligpowht 45W LED Red Blue Lights) photoperiod for approximately 6 weeks before infection with *Agrobacterium rhizogenes.*

### 2.5 Development of stable transgenic hairy root lines

Transgenic *C. roseus* hairy root cultures were generated as previously described (Rizvi et al., 2015). Briefly, six‐week‐old *C. roseus* seedlings (grown as described above) were transformed by pricking the seedling stem above the cotyledons with modified tweezers dipped into the *Agrobacterium rhizogenes* R1000 liquid cultures containing pSB119 (Figure S4). This plasmid encodes the expression of *TurboGFP* (*tGFP*; *Pontellina plumata*) driven by the −914‐bp to +86‐bp *ZCT1* promoter fragment (*pZCT1::tGFP‐I*). Hairy roots emerged from the site of infection approximately ~3 weeks after infection. When hairy roots were longer than 5 mm (~6 weeks after infection), hairy root clusters were excised and cultured first on elimination media to remove *Agrobacterium*, and then on selection media to select for hygromycin‐resistant transgenics. Hairy root cultures were subcultured in liquid media approximately every 28 days and maintained as previously reported (Goklany et al., 2013).

### 2.6 Hormone treatment of stable *pZCT1::tGFP‐I* hairy roots

The *pZCT1::tGFP‐I* hairy roots were cut into pieces approximately 2–3 cm long. Approximately three root pieces were added to each well of 6‐well plates containing 5 ml of liquid Gamborg's media per well and were grown for 7 days. Root pieces were treated with MJ (0.25 or 1 mM; (Goklany et al., 2013)), ABA (0.1, 0.5, or 1 mM; (Wang, Liu, Gao, & Zhang, 2010)), gibberellic acid (GA_3_; 0.01, 0.03, or 0.1 mM; (Suttipanta et al., 2011)), SA (0.01, 0.1, or 0.25 mM (Kang et al., 2004)), or 1‐NAA (0.01, 0.1, or 1 mM (Goddijn, Kam, Zanetti, Schilperoort, & Hoge, 1992)). As some hormones are dissolved in ethanol, a final concentration of 0.1% ethanol was added to all hormone treatments and the mock treatment. The 6‐well plates were incubated in the dark, at 28°C, and shaking for 24 hr. After 24 hr, samples were collected for GFP quantification. Three independent experiments with at least three biological replicates were performed.

### 2.7 Image acquisition

Fluorescence microscopy images were acquired with a Nikon Eclipse 80i microscope and 10x/0.30 Nikon objective (Nikon Corporation), equipped with a SPOT RT3 CCD camera (Diagnostic Instruments), controlled by SPOT Advanced imaging software (v. 5.0) with Peripheral Devices and Quantitative Imaging modules. A Nikon Intensilight C‐ HGFI 130‐W mercury lamp, shuttered with a Lambda 10‐B SmartShutter (Sutter Instruments), was used for GFP excitation, and a GFP filter set (470/40 × 495lpxr 525/50 m; Chroma Technologies) was used for detection.

### 2.8 GFP quantification

Hairy roots expressing TurboGFP were quantified in a fluorescence plate reader after a modified protocol from Marillonnet et al., (2004).

Roots were transferred to filter paper to remove excess culture media. Approximately 100 mg of tissue was transferred into 2‐ml screwcap tubes together with ten 3‐mm glass beads (Fisher), and the fresh weight of the roots was recorded. Samples were frozen in liquid nitrogen and stored at −80°C until analysis. Frozen samples were crushed in a Mini‐BeadBeater‐16 (BioSpec Products Inc.) for 20 s and transferred to ice, and 600 μl of extraction buffer was added (50 mM Na_3_PO_4_‐buffer at pH7.2, 1.0 mM EDTA) with fresh 10 mM β‐mercaptoethanol and 0.1% Triton X‐100. After vortexing and centrifugation (21,000 *g* for 2 min), carefully avoiding cell debris, 200 μl of the supernatant was transferred to a black 96‐well plate and fluorescence was detected with a plate reader (Synergy^TM^ HT, BioTek; excitation 470 nm and emission 509 nm).

### 2.9 Transient *C. roseus* transformation

*Catharanthus roseus* seedlings were transformed with the efficient *Agrobacterium*‐mediated seedling infiltration (EASI) method (Mortensen et al., 2019). Briefly, 10‐day‐old *C. roseus* seedlings were transiently transformed with engineered *Agrobacterium tumefaciens* GV3101 (pMP90) strains by vacuum infiltration, and tissue was collected 3 days after infection. For promoter activity studies, *A. tumefaciens* GV3101 (pMP90) strains, containing the different promoter driving reporter constructs, were adjusted to OD_600_ = 0.2 for infiltration into *C. roseus* seedlings (Figure 4 and 5). Transactivation assays were performed with the reporter strain at OD_600_ = 0.06 and the effector strain at OD_600_ = 0.34 (Figure 6, ratio of reporter to effector strain was optimized in Mortensen et al., 2019) and OD_600_ = 0.4 for transactivation without reporter strain (Figure 7).

### 2.10 Dual‐luciferase assay

FLUC and RLUC activity were analyzed using the Luc‐Pair^TM^ Duo‐Luciferase HT Assay Kit (Genecopoeia) as described in Mortensen et al., (2019).

### 2.11 Monitoring *ORCA3* and *ZCT1* in transiently transformed seedlings

Expression levels of *ORCA3* and *ZCT1* were monitored in seedlings transiently overexpressing a *β‐glucuronidase* (*GUS*) or *ORCA3*, or seedlings lacking *A. tumefaciens* during infiltration, using quantitative real‐time PCR (qRT‐PCR). mRNA was extracted from liquid nitrogen flash‐frozen seedlings (cotyledons from 15 seedlings pooled per biological replicate), stored at −80°C until needed. While still frozen, the cotyledons were crushed by shaking in the Mini‐BeadBeater‐16 (BioSpec) for 15 s with ten 3‐mm glass beads (Fisher). Afterward, RNA was extracted with RNAzol‐RT (Molecular Research Center) and the Direct‐zol RNA Miniprep Plus Kit (Zymo Research) with on‐column DNAse treatment to remove genomic DNA. RNA integrity was assessed using agarose gel electrophoresis, and concentration and purity were quantified with a NanoDrop (ND‐1000 Spectrophotometer; Thermo Scientific). cDNA was synthesized using the SuperScript II First‐Strand Synthesis System (Invitrogen) and oligo‐dT primers with up to 2.5 µg of RNA in a 10 µl reaction, according to the manufacturer's instructions.

cDNA was diluted 1:4, and 1 µl was used in a 10 µl reaction with SYBR Green ROX qPCR Master Mix (Qiagen) on the MX3000P qPCR instrument (Agilent) using the thermocycler protocol previously described with an extension time of 30 s (Goklany et al., 2013). Ct values for each biological replicate were calculated as the average of two technical replicates. Transcript levels were normalized to the housekeeping gene, *SAND* (Pollier, Vanden Bossche, Rischer, & Goossens, 2014), and fold changes were calculated according to the 2^−∆∆Ct^ method (Livak & Schmittgen, 2001). The qPCR primers (Table S1) were designed previously (Goklany et al., 2013; Pollier et al., 2014), but the amplification efficiency for each gene was confirmed again for this study using Ct values over a range of cDNA dilutions and was 100% ± 10% for each gene monitored. No‐reverse‐transcriptase controls were included for each sample, and *SAND* Ct values were confirmed to be at least 5 Ct values above the respective experimental sample (Millipore Sigma technical notes).

## 3 RESULTS

### 3.1 Sequencing and in silico analysis of the *ZCT1, ZCT2*, and *ZCT3* promoters

To begin to characterize the transcriptional regulation of *ZCT1*, the promoter for *ZCT1* was isolated using a genome‐walking approach and the transcription start site (TSS) was determined using 5′ rapid amplification of cDNA ends (RACE). Six clones were sequenced to determine that the TSS is located 86 bp 5′ of the translational start site (ATG). The cDNA ends were the same for 5 of the 6 clones (this is referred to as the TSS in this study), and the 6th included one additional 5′ bp. Our sequence matches the sequence upstream of the *ZCT1* coding region in the sequenced *C. roseus* genomes (Franke et al., 2019; Kellner et al., 2015). *ZCT2* and *ZCT3* are induced with jasmonate like *ZCT1* and have similar functions as *ZCT1* (Chebbi et al., 2014; Goklany et al., 2013; Pauw et al., 2004). Therefore, the promoter sequences of *ZCT2* and *ZCT3* were obtained from the *C. roseus* genome and putative regulatory elements in the promoters of *ZCT1*, *ZCT2,* and *ZCT3* were identified using the Plant Cis‐Acting Regulatory Elements database (PlantCARE) and PlantPAN 3.0. A detailed PlantCARE characterization of the *ZCT1*, *ZCT2*, and *ZCT3* promoters can be found in Figure S1, and motifs identified with PlantPAN 3.0 are included in the supplementary promoter sequences (.gbk files).

The *ZCT1*, *ZCT2,* and *ZCT3* promoters share several common hormone‐ and stress‐responsive elements. All three promoters contain at least one wounding‐responsive element within the first 200 bp upstream from the translational start site. Interestingly, the promoters of *ZCT1* and *ZCT2* both contain two W‐boxes (WRKY binding sites), two abscisic acid‐responsive elements (ABRE), and two ethylene‐responsive elements (ERE). The plant‐specific WRKY TFs (named for the conserved amino acids in WRKY domain) were identified in *C. roseus*, and WRKY1 is involved in regulation of MIA biosynthesis (Schluttenhofer, Pattanaik, Patra, & Yuan, 2014; Suttipanta et al., 2011).

**Figure 1:**
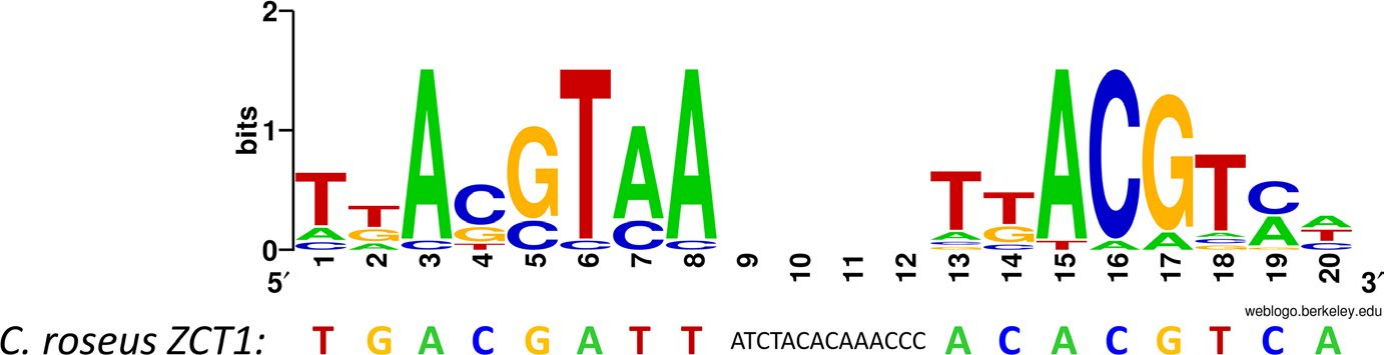
Sequence logo of *as‐1*‐like sequences and *Catharanthus roseus ZCT1 as‐1*‐like sequence. *As‐1*‐like elements were used to create a WebLogo (Crooks et al., 2004). The spacer region (base 9–12 in the WebLogo) was excluded and is usually 4 bp long. The spacer in the *ZCT1* promoter is 14 bp

The *ZCT1* promoter contains multiple elements not found in the other promoters, including motifs involved in gibberellin responsiveness (TATC‐box, pyrimidine box, and GA box, which make up the GA‐responsive complex, GARC; Gubler & Jacobsen, 1992; Lanahan, Ho, Rogers, & Rogers, 1992; Rogers & Rogers, 1992) and two IDD motifs, which are recognized by INDETERMINATE DOMAIN (IDD) proteins and co‐activate with the GA‐responsive protein, DELLA (Yoshida et al., 2014). Additionally, there are two CGTCA or TGACG‐motifs in close proximity, which make up an *activation sequence‐1*‐like element (Krawczyk et al., 2002). We compared the *ZCT1 as‐1*‐like element to well‐characterized *as‐1*‐like elements (Figure 1). The binding sites in the *ZCT1* promoter are consistent with the *as‐1*‐like element, except for the nucleotides at positions 6–8, which are complementary to the expected sequence. Unlike the 4 bp spacers in many plants or *Agrobacterium* genes, or the 6 bp and 9 bp spacers in the *as‐1*‐like elements of the *N. tabacum PR‐1a* and *A. thaliana PR‐1* genes, the *ZCT1 as‐1*‐like element has an uncommonly long 14 bp spacer between the two TGACG‐motifs.

### 3.2 The *ZCT1* promoter responds to several plant hormones

Because we observed several putative hormone‐responsive sites in the *ZCT1* promoter (Figure S1), we explored whether the −914‐bp to +86‐bp fragment of *ZCT1* responds to auxins (1‐NAA), MJ, SA, ABA, and GA_3_. A*s‐1*‐like elements are known to be activated by MJ, SA, and auxins (Ulmasov et al., 1994; Xiang, Miao, & Lam, 1996).

**Figure 2:**
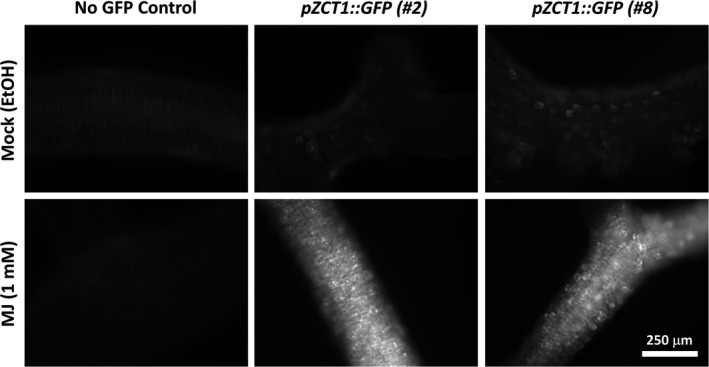
Hairy roots expressing *GFP* under the control of the *ZCT1* promoter show weak basal expression without hormone treatment and increased fluorescence throughout the whole root after treatment with 1 mM MJ. Images show GFP fluorescence of hairy root lines. Hairy roots were treated with ethanol (Mock) or with MJ (1 mM). GFP fluorescence was detected 3 days after the treatment using fluorescence microscopy. The No‐GFP control line lacks a *GFP* gene and shows weak autofluorescence. *pZCT1::GFP* (#2 and #8) are two independent hairy root lines with *GFP* driven by the *ZCT1* promoter

To investigate the transcriptional regulation of *ZCT1*, the −914‐bp to +86‐bp fragment of the *ZCT1* promoter was used to create a reporter with *GFP* (*pZCT1::tGFP‐I*). This vector was used to create stable transgenic hairy root lines via *Agrobacterium*‐mediated transformation as previously described (Rizvi et al., 2015). From eight root lines that passed the hygromycin B selection, two independent lines were chosen for a preliminary experiment to confirm the known responsiveness of *ZCT1* expression with MJ (Goklany et al., 2013; Rizvi, Weaver, Cram, & Lee‐Parsons, 2016). Both lines showed a strong increase in GFP fluorescence throughout the whole root after treatment with MJ (Figure 2). Line #8 was chosen for treatment with various hormones and concentrations.

**Figure 3:**
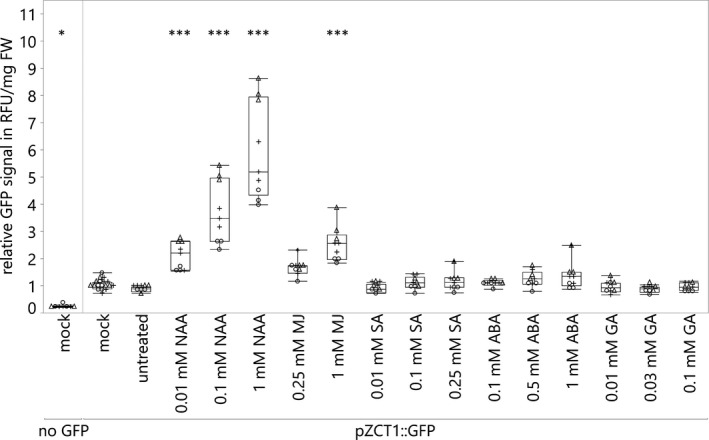
Hairy roots expressing *GFP* under control of the *ZCT1* promoter respond to the auxin 1‐NAA and MJ. Hairy roots expressing *GFP* under the control of the *ZCT1* promoter (*pZCT1::GFP*) or without *GFP* transgene (no *GFP*) were treated with varying concentrations of hormones for 24 hr. The mock treatment contains 0.1% ethanol. No ethanol and no hormones were added to the untreated samples. Each experiment consisted of at least three biological replicates per hormone dosage with a total of three independent assays performed (represented by +, ○, and Δ symbols). For each independent assay, the GFP signal (RFU/mg FW) of each sample is normalized to the average of the *pZCT1::GFP* mock control. Data were analyzed using a one‐way ANOVA, and significant differences, compared to the *pZCT1::GFP* mock control, were determined using the Dunnett's method. *p*‐values < .05 are indicated with one star (*), and *p*‐values < .001 are indicated with three stars (***). NAA, 1‐naphthalene acetic acid; GA, gibberellin (here GA_3_); RFU, relative fluorescence units; FW, fresh weight

Root pieces were treated as described in Materials and Methods. After treatment for 24 hr, GFP expression was quantified using a plate reader. 1‐NAA and MJ induced GFP expression by approximately twofold to sixfold in a dose‐responsive manner, whereas all other hormone treatments (ABA, GA_3_, and SA) resulted in no significant GFP fluorescence change (Figure 3).

To determine whether *pZCT1::tGFP‐I* hairy roots respond to hormones similarly to endogenous *ZCT1,* wild‐type (WT) hairy root cultures were treated with MJ, ABA, and GA_3_ for 7 hr, harvested, and analyzed for *ZCT1* transcript levels using qRT‐PCR. We have previously shown that *ZCT1* expression is responsive to MJ addition by 7 hr (Goklany et al., 2013; Rizvi et al., 2016). In this experiment, *ZCT1* expression showed a dose‐responsive increase with MJ, ABA, and a small decrease with GA_3_ (Figure S2). This response is similar to the response of the *pZCT1::tGFP‐I* hairy root line to MJ and GA_3_. However, increased expression with ABA was not observed in the *pZCT1::tGFP‐I* hairy root line, suggesting that the ABA‐responsive elements might not be captured in the −914‐bp to +86‐bp fragment for the *ZCT1* promoter. Overall, these results suggest that the −914‐bp to +86‐bp fragment of the *ZCT1* promoter contains many of the elements that confer responsiveness of the endogenous gene to hormone treatments such as 1‐NAA and MJ.

### 3.3 The *as‐1‐*like motif contributes to the strong expression from the *ZCT1 *promoter

**Figure 4:**
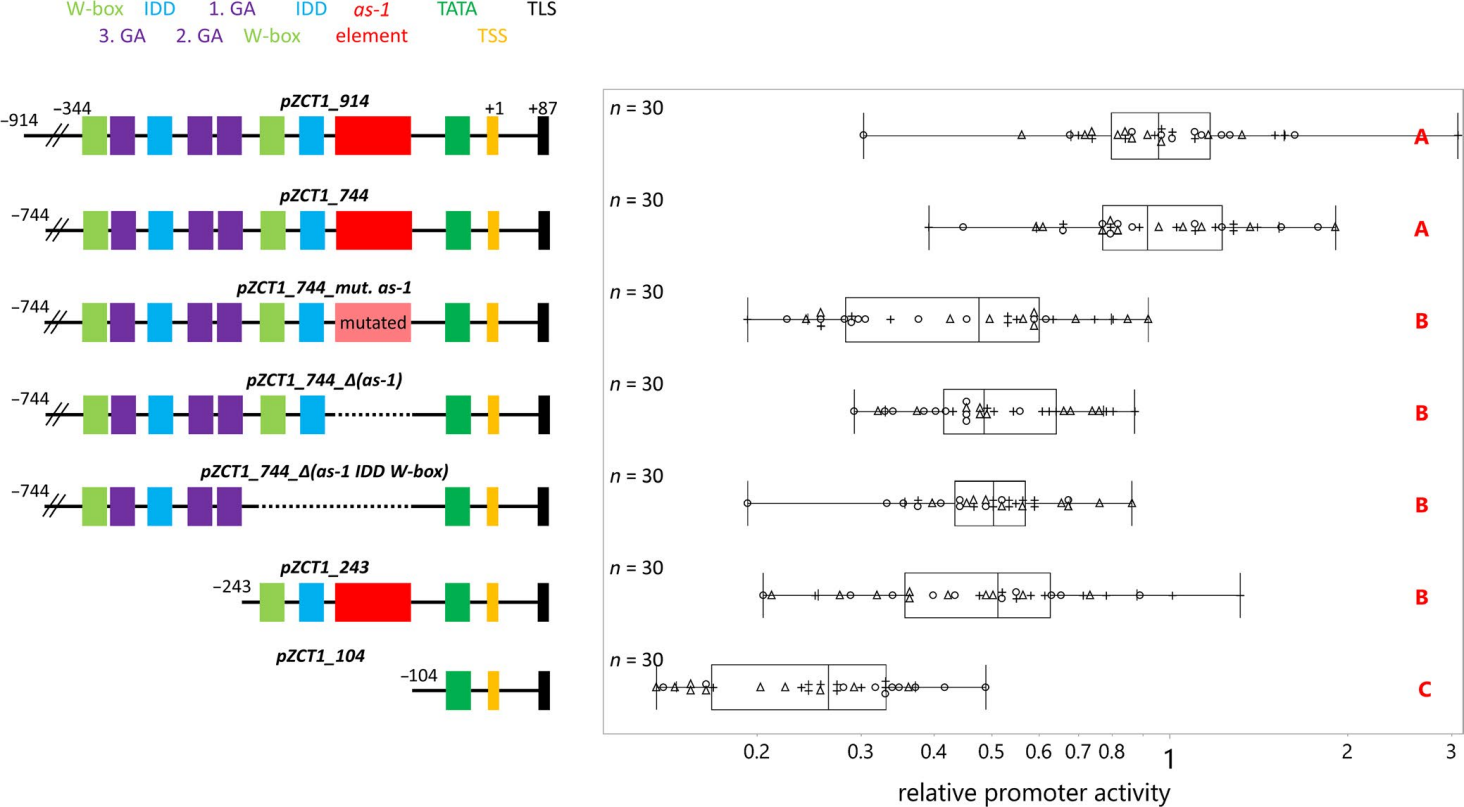
The *as‐1*‐like element is a major contributor to *ZCT1* promoter activity in seedlings. *Catharanthus roseus* seedlings, at three days after transfer to light, were vacuum‐infiltrated with *Agrobacterium tumefaciens* (OD_600_ = 0.2) containing reporter constructs with various *ZCT1* promoter deletion‐driven *FLUC‐I* reporter and the *AtuNOS* promoter‐driven *RLUC‐I* normalization reporter. Samples were taken three days postinfection. The relative promoter activity is the ratio of FLUC (firefly luciferase) to RLUC (*Renilla* luciferase) activity for each sample and then normalized to the ratio of FLUC to RLUC activity of the *pZCT1_744* control (set to 1). The experiment was carried out in three independent assays (represented by +, ○, and Δ symbols). Each data point represents the luciferase activity of 2 seedlings. The vertical line of the boxes shows the median, the ends of the boxes show the 1st and 3rd quantile, and whiskers show the lowest and highest data point values within the 1st and 3rd quartile. Detailed information on the promoter sequence and identified cis regulative elements can be found in Figure S1. The data were log‐normal‐transformed to obtain normal distributed data. Data were analyzed using a one‐way ANOVA, and significant differences between groups were determined using the Tukey–Kramer method on log‐normal‐transformed data and were indicated by different letters

*ZCT1* is expressed at high levels in cotyledons compared to *ORCA3* or the housekeeping gene, *SAND*. During qRT‐PCR experiments, the basal level of *ZCT1* transcripts in cotyledons is high (~20X of SAND), while the basal level of *ORCA3* transcripts is very low (<0.1 of SAND; Figure S3). In order to understand how this high level of expression is conferred, we produced a series of mutant *ZCT1* promoter constructs driving a *FLUC* reporter gene and transiently expressed them in *C. roseus* seedlings using the EASI method (Mortensen et al., 2019), to identify the important elements in the *ZCT1* promoter (Figure 4).

Using PlantCARE and PlantPAN 3.0, a high density of motifs was identified within the first 400 bp of the *ZCT1* promoter (−400 to 0 bp upstream of the TSS). In particular, we identified a region (−350 to −180 bp upstream of TSS) containing a cluster of GA‐responsive elements making up a GA response complex (GARC; TATC‐box, pyrimidine box, GARE) bound by W‐boxes. The GARC bound by W‐boxes is a regulatory unit associated with the antagonistic regulation of GA and ABA in the amylase promoter in rice (Xie et al., 2006). Even though *ZCT1* promoter‐driven *GFP* expression was not increased with GA_3_ in transgenic hairy roots, we hypothesized that the GARC bound by W‐boxes might be differently regulated in seedlings where *ZCT1* was highly expressed (Figure S3). Also, the structure is likely too complex to occur purely by coincidence. Therefore, we chose to further test this cluster in promoter deletion experiments in transiently transformed seedlings. *As‐1‐*like elements are associated with jasmonate (JA) and auxin responsiveness and confer high activity of the promoter such as found in the constitutive cauliflower mosaic virus 35S promoter (Bouchez et al., 1989; Liu & Lam, 1994). The *as‐1*‐like element was chosen for transient promoter deletion experiments, as the *pZCT1::GFP* expression in transgenic hairy roots was induced by JA and auxin.

The two longest *ZCT1* promoter fragments (−914 bp to +86 bp, *pZCT1_914*, or −744 bp to +86 bp, *pZCT1_744*) provide similar levels of expression (Figure 4). The *pZCT1_914* construct contains several additional stress response elements (STRE), but these do not significantly increase the promoter activity compared to the *pZCT1_744* construct under the tested conditions.

To test the importance of the *as‐1*‐like element, a 58 bp nucleotide deletion (*pZCT1_744_ Δ(as‐1)*) of the *as‐1*‐like element and a mutant construct (*pZCT1_744_mut. as‐1*) were expressed using the EASI method. If the *as‐1*‐like element is mutated or deleted (*pZCT1_744_mut. as‐1* or *pZCT1_744_ Δ(as‐1)*), promoter activity is reduced by approximately 50%. If one IDD binding site as well as one W‐box is removed in addition to the *as‐1*‐like element (*pZCT1_744_ Δ(as‐1 IDD W‐box)*), activity is not reduced compared to the deletion of just the *as‐1*‐like element (*pZCT1_744_ Δ(as‐1)*), suggesting that this W‐box is not contributing significantly to the *as‐1*‐like‐driven promoter activity under the given conditions. A 501 bp 5′ deletion (*pZCT1_243)* also results in a reduction of promoter activity by approximately 50%. This section contains multiple elements, including the GARC. The *pZCT1_104* fragment, which contains the TATA‐box and transcription start site alone, confers ~26% of the promoter activity, suggesting that there are additional regulatory elements within this 104‐bp fragment.

**Figure 5:**
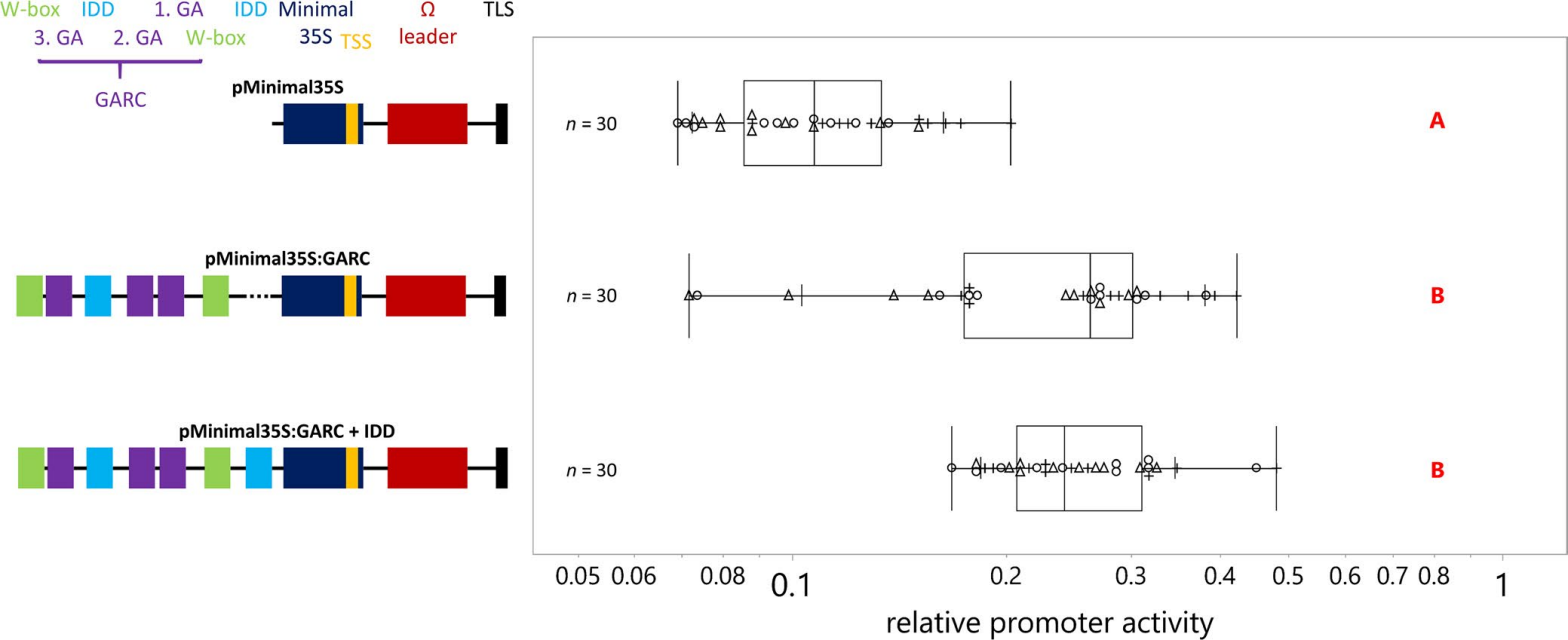
The GA‐responsive complex (GARC) is a contributor to *ZCT1* promoter activity under the EASI conditions. *Catharanthus roseus* seedlings, at three days after transfer to light, were vacuum‐infiltrated with *Agrobacterium tumefaciens* (OD_600_ = 0.2) containing reporter constructs with various *ZCT1* promoter deletion‐driven *FLUC‐I* reporter and the *AtuNOS* promoter‐driven *RLUC‐I* normalization reporter. Samples were taken three days postinfection. The relative promoter activity is the ratio of FLUC (firefly luciferase) to RLUC (*Renilla* luciferase) activity for each sample normalized to the ratio of FLUC to RLUC activity of the *pZCT1_744* control (set to 1). The experiment was carried out in three independent assays (represented by +, ○, and Δ symbols). Each data point represents the luciferase activity of 2 seedlings. The vertical line of the boxes shows the median, the ends of the boxes show the 1st and 3rd quantile, and whiskers show the lowest and highest data point values within the 1st and 3rd quartile. Detailed information on the promoter sequence and identified cis regulative elements can be found in Figure S1. The data were log‐normal‐transformed to obtain normal distributed data. Data were analyzed using a one‐way ANOVA, and significant differences between groups were determined using the Tukey–Kramer method on log‐normal‐transformed data

Next, we investigated the GARC without the overshadowing effect of the *as‐1*‐like element (Figure 5). Fusion of the GARC (including the two IDD binding sites and the W‐boxes) to the 35S minimal promoter (*pMinimal35S:GARC + IDD*) significantly increases the promoter activity of the 35S minimal promoter. Deletion of one IDD binding site from this fragment (*pMinimal35S:GARC*) does not significantly affect the activity of the *pMinimal35S:GARC + IDD* promoter fragment. These results show that the GARC confers promoter activity under the tested conditions, and that the second IDD binding site does not synergistically enhance promoter activity. However, overall, the promoter activity of the 35S minimal promoter fusions is quite low compared to the *pZCT1_744* activity.

Taken together, these results suggest the *as‐1*‐like element contributes significantly to the expression of *ZCT1* in seedlings under the EASI transformation conditions. Other elements in the promoter, including the GARC, also contribute to *ZCT1* expression.

### 3.4 ORCA3 does not transactivate the *ZCT1* promoter, while ZCT1 represses its own promoter

**Figure 6:**
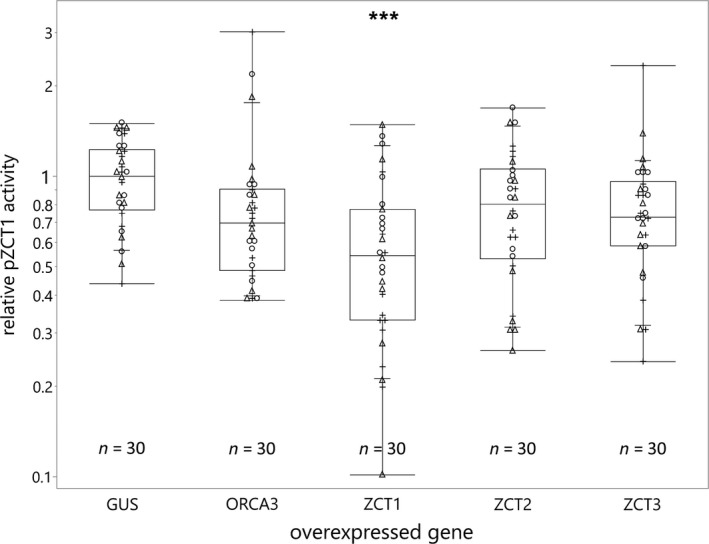
ORCA3 does not activate the *ZCT1* promoter, but ZCT1 represses its own promoter. *Catharanthus roseus* seedlings, at three days after transfer to light, were vacuum‐infiltrated with a combination of two strains of *Agrobacterium tumefaciens* (total OD_600_ = 0.4): (I) strain containing the *ZCT1* promoter‐driven *FLUC‐I* reporter and the *AtuNOS* promoter‐driven *RLUC‐I* normalization reporter (plasmid pSB135; OD_600_ = 0.06), and II) strain containing a *CaMV2x35s*‐driven effector for transactivation (*GUS* as control—pSB161 (Addgene ID #123197), *ORCA3*—pSB160 (Addgene ID #123196), or *ZCTs*—pSB153‐155; OD_600_ = 0.34). Samples were taken three days postinfection. The *ZCT1* promoter activity is the ratio of FLUC (firefly luciferase) to RLUC (*Renilla* luciferase) activity for each sample normalized to the ratio of FLUC to RLUC activity of the GUS control (set to 1). The experiment was carried out in three independent assays (represented by +, ○, and Δ symbols). Each data point represents luciferase activity of 2 seedlings. The horizontal line of the boxes shows the median, the ends of the boxes show the 1st and 3rd quantile, and whiskers show the lowest and highest data point values within the 1st and 3rd quartile. The data were log‐normal‐transformed to obtain normally distributed data. Data were analyzed using a one‐way ANOVA, and significant differences, compared to the GUS control, were determined using the Dunnett's method on log‐normal‐transformed data. *p*‐values < .001 are indicated with three stars (***)

**Figure 7:**
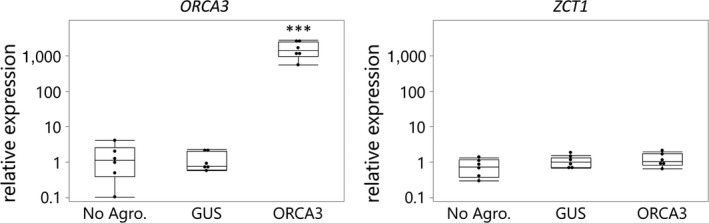
*ORCA3* overexpression does not increase *ZCT1* expression, and *ZCT1* is not significantly induced under the EASI conditions using *Agrobacterium tumefaciens* strain GV3101. *Catharanthus roseus* seedlings were transiently transformed, as described in Mortensen et al., (2019), with *Agrobacterium tumefaciens* containing either a *GUS* (Addgene ID #123197) or *ORCA3* (Addgene ID #123196) overexpression construct (Figure S4). The “No Agro.” condition was treated identical to the other infiltrations but with no *Agrobacteria* present. Transcript levels were normalized to the housekeeping gene, *SAND* (Pollier et al., 2014), and fold changes were calculated according to the 2^−∆∆Ct^ method relative to the *GUS* control (Livak & Schmittgen, 2001). Data were analyzed using a one‐way ANOVA, and significant differences, compared to the *GUS* control, were determined using the Dunnett's method. *p*‐values < .001 are indicated with three stars (***)

Previously, elevated *ORCA3* expression levels were correlated with increased expression of *ZCT1*, *ZCT2,* and *ZCT3* in a stable hairy root line, suggesting the regulation of *ZCTs* by ORCA3 (Peebles et al., 2009). Using the EASI method, we investigated the transactivation of the *ZCT1* promoter (pSB135, *pZCT1_914::FLUC‐I*) by either one of the transcription factors (ORCA3, ZCT1, ZCT2, or ZCT3) or GUS (control) in *C. roseus* seedlings. The co‐expression of the *ORCA3* and the promoter–reporter construct did not result in the activation of the *ZCT1* promoter (Figure 6). Overexpression of *ORCA3* also did not induce *ZCT1* mRNA levels (Figure 7). In comparable experiments, ORCA3 was able to transactivate the *STR1* promoter by approximately 10‐fold (Mortensen et al., 2019). These data suggest that the previously observed regulation of *ZCT1* by ORCA3 in hairy roots (Peebles et al., 2009) may be indirect or root‐specific. In addition, ZCT1 significantly represses its own promoter (~ 40%), while no significant effect is observed with ZCT2 or ZCT3 (Figure 6). This result suggests a possible mechanism for autoregulation of *ZCT1* expression by ZCT1.

## 4 DISCUSSION

In plants, Cys_2_/His_2_‐type (C_2_H_2_) zinc finger proteins such as ZCT1 are an important class of transcription factors involved in controlling growth, development, and stress‐responsive genes (cold, drought, salt, and oxidative stress; Ciftci‐Yilmaz & Mittler, 2008; Kiełbowicz‐Matuk, 2012). They contain an EAR‐motif involved in active repression (Ciftci‐Yilmaz et al., 2007; Hiratsu, Matsui, Koyama, & Ohme‐Takagi, 2003). Despite their importance, the knowledge is limited on the regulatory networks that control the expression of these factors in response to stresses.

Here, we showed that the promoter of *ZCT1* contains several hormone‐responsive elements. Some of these elements are shared in the promoters of other *ZCT* genes in *C. roseus*, *ZCT2* and *ZCT3*. However, the *C. roseus ZCT1* promoter is particularly interesting due to the high number and arrangement of elements (Figure S1). These in silico observations suggest that ZCT1 may be involved in the crosstalk between phytohormone and defense signaling pathways.

However, little has been shown regarding the hormonal regulation of *ZCT1* expression. We previously showed that *ZCT1* expression was strongly induced with high dosages of MJ (1 mM) in hairy root cultures (Goklany et al., 2013). Here, we showed that the −914‐bp to + 86‐bp region upstream of *ZCT1* also leads to strong induction with auxin (1‐NAA) (Figure 3), as well as with MJ in transgenic hairy roots. ABA also induced *ZCT1* expression, but potentially through elements outside of the −914‐bp to + 86‐bp upstream region tested in the transgenic hairy roots (Figure S2).

The responsiveness of *ZCT1* expression with high auxin levels suggests their role in decreasing MIA production under growth‐promoting conditions. The synthetic auxin, 2,4‐dichlorophenoxyacetic acid (2,4‐D), repressed the hydroxymethylbutenyl 4‐diphosphate synthase (*HDS*) gene from the methyl erythritol phosphate (MEP) pathway (Chebbi et al., 2014). The MEP pathway provides isopentenyl diphosphate (IPP) for MIA biosynthesis. ZCT2 was pulled down in a yeast one‐hybrid assay with a region from the *HDS* promoter and both ZCT1 and ZCT2 repressed the *HDS* promoter (Chebbi et al., 2014). These results by Chebbi et al. (2014) support our hypothesis that the induction of * ZCT1 *promoter activity by auxin is leading to a repression of MIA synthesis. Additionally, the responsiveness of the *ZCT1* promoter to auxins explains why *ZCT1* levels were increased during transient transformation of *C. roseus* seedlings with *A. rhizogenes* strain R1000 (Weaver, Goklany, Rizvi, Cram, & Lee‐Parsons, 2014), which transfers genes for auxin biosynthesis into plants (Inzé et al., 1984). *ZCT1* levels were not increased during transient transformation of *C. roseus* seedlings with the *A. tumefaciens* strain GV3101, which has been disarmed of its endogenous plant hormone biosynthetic genes (Figure 7).

The strong induction of *ZCT1* promoter activity with auxin and MJ may be due to the *as‐1*‐like element (Figure 1); *as‐1*‐like elements are observed to be responsive to auxins, MJ, SA, and further stressors (Ulmasov et al., 1994; Xiang et al., 1996). Initially, it was surprising to us that the *pZCT1::GFP* line did not respond to treatment with SA (Figure 3), but Van der Does et al. (2013) showed that W‐boxes (which are present in the * ZCT1 *promoter (Figure 4 and Figure S1)) are enriched in promoter regions of MJ‐inducible genes that are antagonized by SA. This suggests a possible mechanism for repression of MJ‐induced promoter activity by SA‐induced *WRKY* expression and binding to W‐boxes.

The importance of the *as‐1*‐like element for *ZCT1* promoter activity was shown by the promoter deletions studies in seedlings (Figure 4), as ~50% of the promoter activity was lost if the *as‐1*‐like element was deleted or mutated. To demonstrate its activity in the absence of the *as‐1‐*like element, the GARC was fused to the 35S minimal promoter and conferred increased promoter activity in seedlings (Figure 5). However, GA_3_ did not induce *GFP* expression in hairy roots (Figure 3); these potential discrepancies could be attributed to a condition‐specific role of the GARC. For instance, in rice aleurone cells, the expression of the amylase gene is regulated by the GARC cluster bounded by W‐boxes, promoting the breakdown of starch in germinating seedlings in the presence of GA_3_ (Xie et al., 2006).

Peebles et al. (2009) observed increased *ZCT1* expression when *ORCA3* was overexpressed in hairy roots, suggesting a possible regulation of *ZCT1* through ORCA3. We did not observe the induced expression of *ZCT1* when *ORCA3* was overexpressed in seedlings (Figure 7) nor the transactivation of *ZCT1* promoter activity by ORCA3 (Figure 6). This suggests that the overexpression of *ORCA3* alone is not sufficient to induce *ZCT1*. Interestingly, we observed a repression of *ZCT1* on its own promoter (Figure 6), suggesting a possible feedback regulation for limiting *ZCT1* expression. Key MIA pathway activators, like *BIS* and *ORCA*, have been shown to have a positive feedback regulation (Schweizer et al., 2018). The *as‐1*‐like element had the strongest effect on *ZCT1* promoter activity in seedlings (Figure 4). *As‐1*‐like elements are known to be regulated through the TGA family of basic‐leucine‐zipper (bZIP) transcription factors (Lam & Lam, 1995), providing a target group for further investigation of regulators of the *ZCT1* promoter.

In summary, we have identified *ZCT1* promoter activity as responsive to auxin (1‐NAA) and MJ. An *as‐1*‐like element is particularly important for promoter activity and suggests a positive regulation of *ZCT1* through TGA transcription factors. In contrast to initial expectations, ORCA3 did not transactivate the *ZCT1* promoter, while ZCT1 represses its own promoter. Future investigation into *ZCT1* regulation will provide insights into the important biological problem of how phytohormone crosstalk and/or feedback mechanisms are integrated at the level of transcription of regulatory proteins.

## CONFLICT OF INTEREST

The authors declare that they have no conflict of interest.

## AUTHOR CONTRIBUTIONS

SM, JW, EJC, and CL‐P conceived and designed the research. SM, SS, JW, LC, and NR performed the experiments. SM, SS, JW, LC, NR, EJC, and CL‐P analyzed the data. SM, JW, EJC, and CL‐P wrote the manuscript.
