# Supplementary material for: The regulation of ZCT1, a transcriptional repressor of monoterpenoid indole alkaloid biosynthetic genes in Catharanthus roseus
Source: Plant Direct. 2019 Dec 31;3(12):e00193. doi: 10.1002/pld3.193 (PMC6937483; doi:10.1002/pld3.193)
Supplement: Supplementary file 1 [file PLD3-3-e00193-s001.pdf]

Supplemental Materials

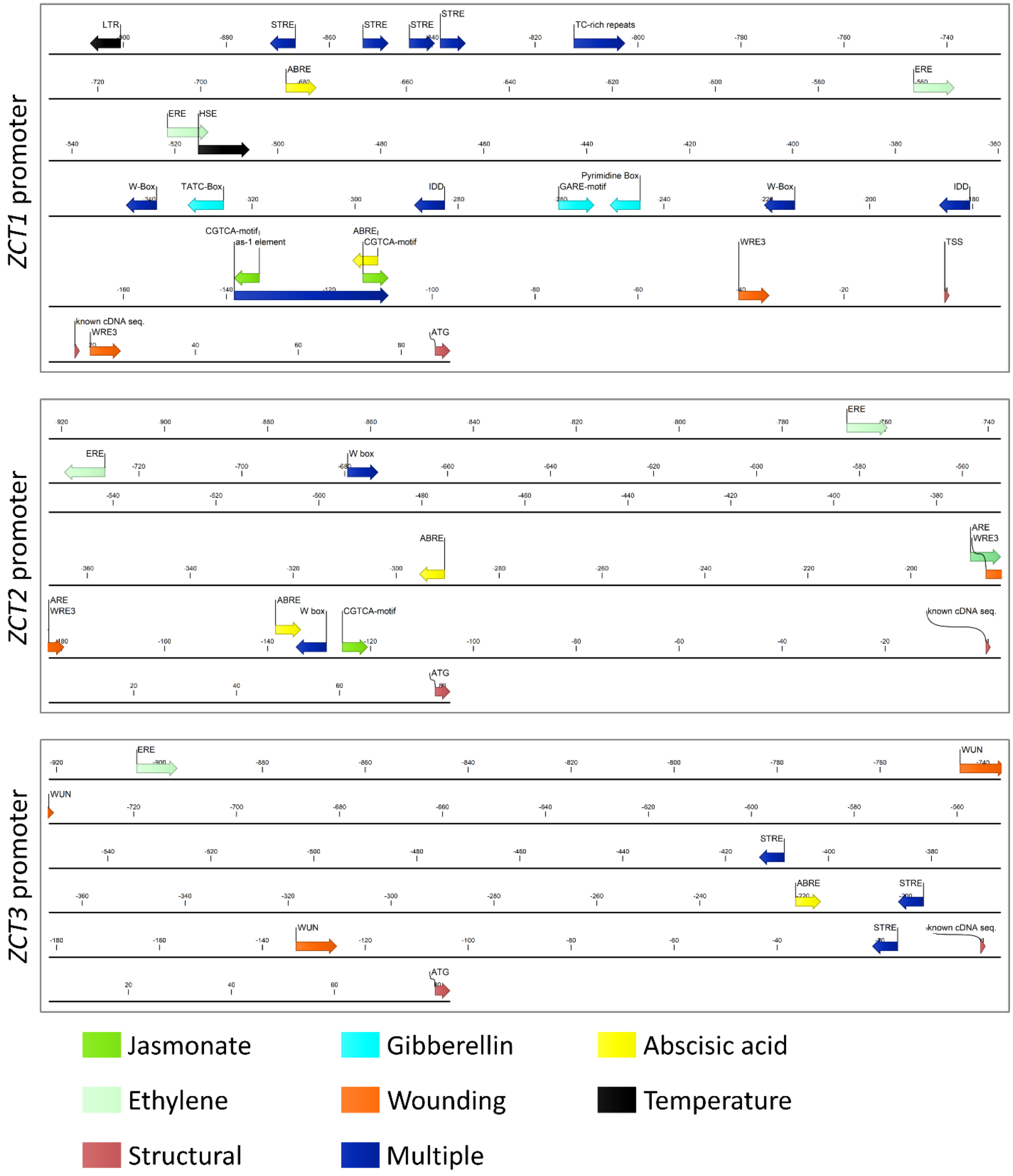

Figure S1 Detailed *in silico* analysis of the *C. roseus ZCT1*, *ZCT2*, and *ZCT3* promoters.

Schematic illustrating putative hormone- and stress-responsive regulatory elements found in the promoters of *ZCT1*, *ZCT2*, and *ZCT3*. Analysis was limited to 1kb upstream of the start codon. Key promoter elements: Jasmonate (JA): CGTCA-motif; Gibberellin (GA): TATC-Box, gibberellin responsive element (GARE)-motif, and the pyrimidine box make up a GA responsive complex (GARC); Abscisic acid (ABA): abscisic acid responsiveness (ABRE); Ethylene: ethylene-responsive element (ERE); Wounding: wounding responsive element (WRE3 and WUN); Temperature: low-temperature responsiveness (LTR), heat stress responsiveness (HSE); Structural: transcriptional start site (TSS), known cDNA sequence, start codon (ATG); Multiple: stress response element (STRE), defense and stress responsiveness (TC-rich repeats), W-box respond to biotic and abiotic stress, IDD-binding sequence (IDD), an *as-1*-like element made up by two CGTCA-motifs in close proximity. Light responsive elements were excluded.

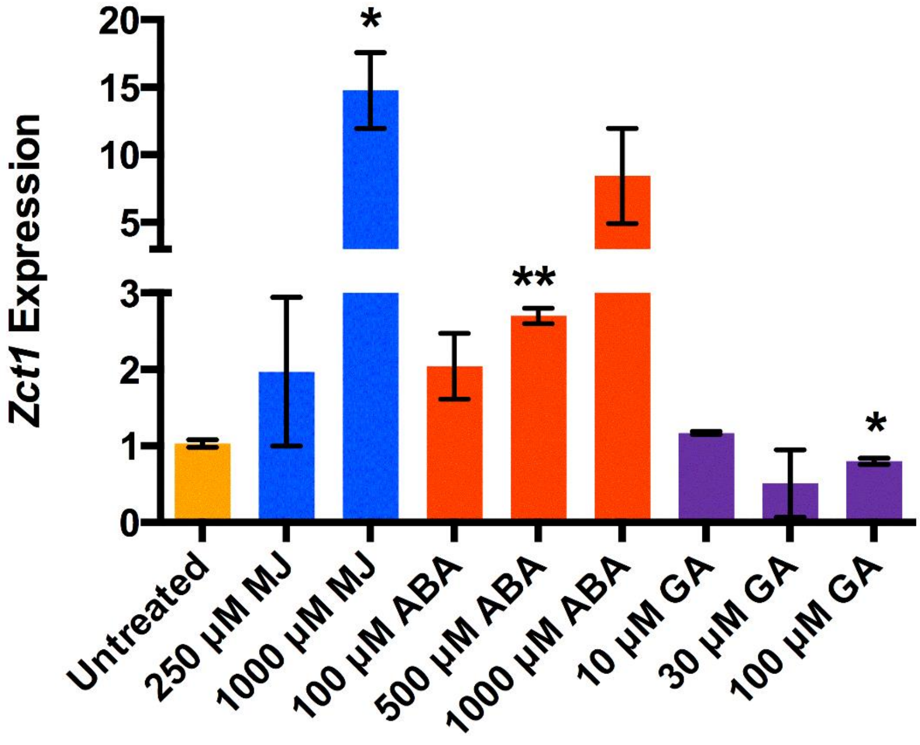

Figure S2 *ZCT1* responds in hairy roots to MJ and ABA

ZCT1 expression level determined by qRT-PCR in a stable WT hairy root line treated as indicated for 7 h (n=2). Transcript levels were normalized to the housekeeping gene, *Rsp9*, and fold changes were calculated according to the $2^{-\Delta\Delta Ct}$ method relative to the untreated condition (Livak & Schmittgen, 2001). The Student's t test was used to calculate statistical significance relative to the untreated samples. Error bars represent standard deviations and * denotes $p < 0.05$ and ** denotes $p < 0.01$.

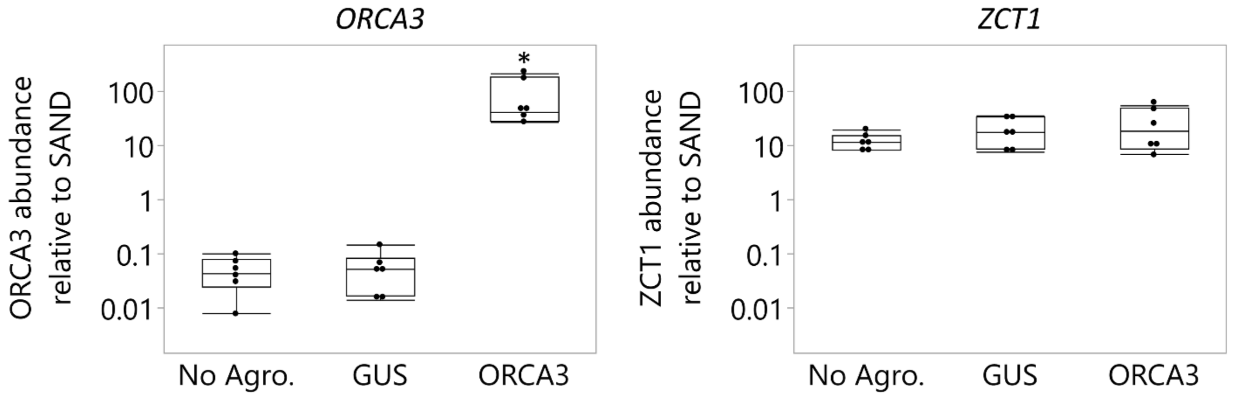

Figure S3 ***ZCT1*** **is highly expressed, while** ***ORCA3*** **is low expressed, in seedlings**

C. roseus seedlings were transiently transformed, as described in Mortensen *et al.*, 2019, with *A. tumefaciens* containing either a *GUS* (Addgene ID #123197) or *ORCA3* (Addgene ID #123196) overexpression construct (Figure S4). The “No Agro.” condition was treated identical to the other infiltrations but with no *Agrobacteria* present. Relative abundance of the gene of interest (GOI) is the $2^{Ct\ SAND\text{-}Ct\ GOI}$, with SAND as the reference gene. Data was analyzed using a one-way ANOVA and significant differences, compared to the *GUS* control, were determined using the Dunnett’s method. P-values <0.05 are indicated with one star (*).

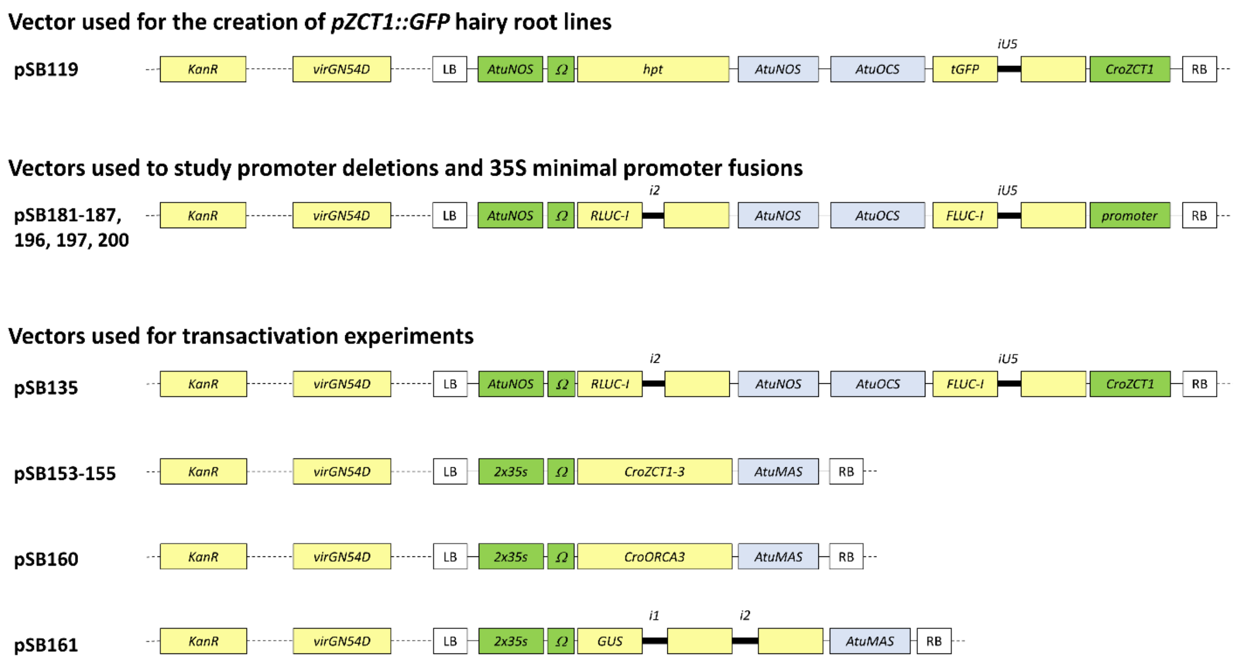

Figure S4 Binary vectors used in this study

Promoters including 5'UTRs are shown in green, coding sequences in yellow, and terminators in blue. Introns are indicated with a black bar within the coding sequence. The vectors pSB153, pSB160 and pSB161 have been described previously (Mortensen et al., 2019). All other vectors were newly constructed in this study with parts from Weber *et al.*, 2011, Engler *et al.*, 2014, and Mortensen *et al.*, 2019 or with new parts described in this study. For the vector backbones pSB90 was used (Mortensen et al., 2019).

Supplementary Table 1: Primers sequences (5'-3')

Primers for amplifying *ZCT1* Promoter with genome walking	
*ZCT1*PGSP1*	CAACGCCGGTACTGCTGATTTCTCCAT
*ZCT1*PGSP2*	GCGGATTGTTCTTCTCTGAATCTCTCT
*ZCT1*PGSP3	CAGCGGAAAAACTAACATGC
*ZCT1*PGSP4	CTACGCATAATATGTACCAGCCCAG
*ZCT1*P(-1961)	TCGTTCCACTGAGCGTCAGACC
Primers used for creating pDONR221-*pZCT1* deletions	
(deletions were first created in pDONOR before moving them into the MoClo system)	
*ZCT1*P(-243)F	GAGAAGGTTCCTAGTTGAGGAAG
*ZCT1*P(-769)R	CTACGCATAATATGTACCAGCCCAG
*ZCT1*P(-104)F	CACCTCTCTCTCTGCTTTCTC
*ZCT1*P(94)F	GAAGAGATTCAGAGAAGAACAATCCGC
*ZCT1*P(-162)R	GAGTAAGGGAGGGAAGATAGAC
*ZCT1*P(-243)R	CTTCCTCAACTAGGAACCTTCTC
Primers used for cloning the *ZCT1* promoter for pSB119, pSB135, pSB187	
Pzct1-1000_F	ttgaagacaaGGAGTGTCTCATTTTCGGAGTTTAGCTTGC
Pzct1-1_R	ttgaagacaaCATTGTTTTTTGTTATTGAATTGAAACTTGCGAC
Primers used for cloning the *ZCT2* coding sequence (pSB154)	
ZCT2_mo_F1	ttgaagacaaAATGGTGATGATTAATATACCGATGAAGC
ZCT2_mo_R1	ttgaagacaaCTCCTCATATGACCTCCGAGAGC
ZCT2_mo_F2	ttgaagacaaGGAGACATAGGGCGGCGATG
ZCT2_mo_R2	ttgaagacaaAAGCTCATAAGAAGCAATCAACAGTTGG
Primers used for cloning the *ZCT3* coding sequence (pSB155)	
ZCT3_mo_F	ttgaagacaaAATGGCACTTGAAGCTTTGAATTC
ZCT3_mo_R	ttgaagacaaAAGCCTAATTAATTTGATGGTTTTCAACTTTGATC
Primers used for creating *pZCT1* deletions in MoClo (pSB181-187)	
ZCT1prom_R_MoClo	ttgaagacaaCATTGTTTTTTGTTATTGAATTGAAACTTGC
Del1_F_MoClo	ttgaagacaaGGAGGAGAAGGTTCCTAGTTGAGGAAGAG
Del2_F_MoClo	ttgaagacaaGGAGCACCTCTCTCTCTGCTTTCTCTTTC
Del4/5/mutAs-1_F_MoClo	ttgaagacaaGGAGGATTACTGCGATTAATTAGGATATTACCTAATG
Primers used for creating 35S minimal fusions in MoClo (pSB196, 197, 200)	
35S_R_BpiI_mis	TAAGGgaagacCCCATTGTATCG
primer151	ttgaagacaaGGAGGCAAGACCCTTCCTCTATATAAG
primer153	ttgaagacaaTACTGCAAGACCCTTCCTCTATATAAG
primer154	ttgaagacaaGGAGTAGAGTAGTCAAAATCTATGGGATAAAAAAG
primer155	ttgaagacaaAGTACCTTGTTTGACTCTTCCTCAAC
primer156	ttgaagacaaAGTAGATAGACAAGGATTCATAAGAAAGGC
Primers for transiently transformed seedlings and WT hairy roots qRT-PCR analysis	
Rps9_F	TCCACCATGCCAGAGTGCTCATTA
Rps9_R	TCCATCACCACCAGATGCCTTCTT
SAND_qF	TGCTGTGGAGGAGGAAGAAG
SAND_qR	ACTGGCGGAACTACTACTACC

ZCT1_F	AATCTTTAGCGGTGACGAAGCCGA
ZCT1_R	CGTTGTCCTCAGGCGTCAAATTCA
Orca3-forward	TGTCAGGAGGATTCTGTTGTGGGA
Orca3-reverse	CGCATATTAAACGCGGCTGCATCA

*****Also used in 5’RACE.

Monitoring *ZCT1* expression in HRs using qRT-PCR

To determine endogenous response levels, WT hairy roots were treated with some chosen hormone concentrations for 7 h, flash-frozen, and qRT-PCR analysis of *ZCT1* expression was performed as described below.

Stable WT hairy roots treated with hormones were harvested and ground in liquid nitrogen, and mRNA was extracted using the RNAzol®RT method. cDNA was synthesized from the mRNA using the SuperScript First-Strand Synthesis System for RT-PCR (Invitrogen) as previously described (Goklany, Loring, Glick, & Lee-Parsons, 2009). *ZCT1* expression levels were monitored using the primers listed in Supplementary Table 1. qRT-PCR was performed using the RT^2 Real-Time™ SYBR Green PCR master mix (SABiosciences) and the Bio-Rad iQ5 Real-Time PCR using the thermocycler protocol as previously described (Goklany et al., 2009). *Rps9* was used as the housekeeping gene (Collu et al., 2001; Menke, Champion, Kijne, & Memelink, 1999; Van der Fits & Memelink, 2000). The fold increase for *ZCT1* was calculated using the ΔΔCt method relative to untreated WT hairy roots, denoted with a fold change of 1.0 (Livak & Schmittgen, 2001).

References

Collu, G., Unver, N., Peltenburg-Looman, A. M. G., Van der Heijden, R., Verpoorte, R., & Memelink, J. (2001). Geraniol 10-hydroxylase, a cytochrome P450 enzyme involved in terpenoid indole alkaloid biosynthesis. *FEBS Letters*, *508*(2), 215–220. https://doi.org/10.1016/S0014-5793(01)03045-9

Engler, C., Youles, M., Gruetzner, R., Ehnert, T. M., Werner, S., Jones, J. D. G., … Marillonnet, S. (2014). A Golden Gate modular cloning toolbox for plants. *ACS Synthetic Biology*, *3*(11), 839–843. https://doi.org/10.1021/sb4001504

Goklany, S., Loring, R. H., Glick, J., & Lee-Parsons, C. W. T. (2009). Assessing the limitations to terpenoid indole alkaloid biosynthesis in catharanthus roseus hairy root cultures through gene expression profiling and precursor feeding. *Biotechnology Progress*, *25*(5), 1289–1296. https://doi.org/10.1002/btpr.204

Livak, K. J., & Schmittgen, T. D. (2001). Analysis of relative gene expression data using real-time quantitative PCR and the 2-ΔΔCT method. *Methods*, *25*(4), 402–408. https://doi.org/10.1006/meth.2001.1262

Menke, F. L. H., Champion, A., Kijne, J. W., & Memelink, J. (1999). A novel jasmonate- and elicitor- responsive element in the periwinkle secondary metabolite biosynthetic gene Str interacts with a jasmonate- and elicitor- inducible AP2- domain transcription factor, ORCA2. *The EMBO Journal*, *18*(16), 4455–4463. https://doi.org/10.1093/emboj/18.16.4455

Mortensen, S., Bernal-Franco, D., Cole, L. F., Sathitloetsakun, S., Cram, E. J., & Lee-Parsons, C. W. T. (2019). EASI Transformation: An Efficient Transient Expression Method for Analyzing Gene Function in Catharanthus roseus Seedlings. *Frontiers in Plant Science*,

10(June), 1–17. https://doi.org/10.3389/fpls.2019.00755

Van der Fits, L., & Memelink, J. (2000). ORCA3, a Jasmonate-Responsive Transcriptional Regulator of Plant Primary and Secondary Metabolism. *Science*, *295*(July), 295–297. https://doi.org/10.1126/science.289.5477.295

Weber, E., Engler, C., Gruetzner, R., Werner, S., & Marillonnet, S. (2011). A modular cloning system for standardized assembly of multigene constructs. *PLoS ONE*, *6*(2). https://doi.org/10.1371/journal.pone.0016765
