# Supplementary material for: The regulation of ZCT1, a transcriptional repressor of monoterpenoid indole alkaloid biosynthetic genes in Catharanthus roseus
Source: Plant Direct. 2019 Dec 31;3(12):e00193. doi: 10.1002/pld3.193 (PMC6937483; doi:10.1002/pld3.193)
Supplement: Supplementary file 2 [file PLD3-3-e00193-s002.pdf]

Dear Dr Farre,

Dear Dr Fortes,

Dear Reviewer,

Thank you for carefully reviewing our manuscript and thank you for providing your feedback. We appreciate your work and help.

Below are your comments and our responses (in blue). We hope that we have addressed all your comments to your satisfaction. Line numbers are referring to the version with track changes (all markup).

Thank you.

~~~~~~

Reviewer comments:
Reviewer #1:

This manuscript describes a study of regulatory interactions with the promoter of the transcription factor ZCT1, performed in the medicinal plant *C. roseus* where ZCT1 plays a key role in biosynthetic pathways for valued compounds. The article makes three primary claims and provides supporting evidence for each claim:

1) The presence of a particular motif called as-1-like-motif in the ZCT1 promoter significantly affects ZCT1 expression in *C. roseus* seedlings:
Transient expression assays in seedlings are used to show that ZCT1 promoter constructs where the as-1-like element has been mutated or deleted drive a FLUC reporter at substantially lower levels than control constructs.

2) The transcriptional activator ORCA3 doesn't upregulate ZCT1 expression in seedlings when ORCA3 is transiently over-expressed in seedlings:
ORCA3, and GUS as a control, were both overepressed and monitored in seedlings using qRT-PCR; there was no observable activation of the ZCT1 promoter or increase in ZCT1 mRNA levels.

3) ZCT1 represses its own promoter region (whether the mechanism is direct or indirect is not evaluated):
~~~~~~

When ZCT1 was overexpressed and monitored with control, ZCT1 promoter activity decreased. (Direct binding of ZCT1 to its own promoter is not assayed.)

Each experiment is performed with appropriate controls, and the evidence provided does solidly support the claims.

A few minor suggestions for improvement of the manuscript:

Lines 45-46: Should the statement on these lines have a citation?

Response: We added the references Pauw et al., 2004 and Goklany et al., 2013 (line 49).

Line 239: Addition of a hyphen would clarify:
"No reverse-transcriptase controls were included for each sample,"
-> No-reverse-transcriptase controls

Response: Added a hyphen (line 256).

Line 257: Figures are first mentioned here starting with Figure 4. Should the figures be re-numbered to match the order in which they are addressed in the manuscript?

Response: We removed the reference to Figure 4 as it is not essential at this point (lines 273-274).

Line 317-320: I believe the statements in these lines continue to refer to Figure 4, but because this is a new paragraph and Figure 4 isn't directly referenced this gets a little confusing. Addition of (Figure 4) to at least the first statement in this new paragraph would help clarify.

Response: We added the references to Figure 4 to the first sentence of this paragraph (line 352).

Line 322: It would be helpful to the reader to clarify here: Why among the several important regulatory elements are as-1-like and GARC specifically selected for testing?

Response: We added the lines below, explaining the reasoning why we in particular chose the GARC and the as-1-like sequence for further evaluation (lines 336-350).

"Using PlantCARE and PlantPAN 3.0, a high density of motifs was identified within the first 400 bp of the *ZCT1* promoter (-400 to 0 bp upstream of the TSS). In particular, we identified a region (-350 to -180 bp upstream of TSS) containing a cluster of GA-responsive elements making up a GA response complex (GARC; TATC-box, pyrimidine box, GARE) bound by W-boxes. The GARC bound by W-boxes is a regulatory unit associated with the antagonistic regulation of GA and ABA in the amylase promoter in rice (Xie et al., 2006). Even though *ZCT1* promoter driven GFP expression was not increased with GA_3 in transgenic hairy roots, we hypothesized that the

GARC bound by W-boxes might be differently regulated in seedlings where *ZCT1* was highly expressed (Figure S3). Also, the structure is likely too complex to occur purely by coincidence. Therefore, we chose to further test this cluster in promoter deletion experiments in transiently transformed seedlings. As-1-like elements are associated with jasmonate (JA) and auxin responsiveness and confer high activity of the promoter such as found in the constitutive cauliflower mosaic virus 35S promoter (Bouchez et al., 1989; Liu and Lam, 1994). The *as-1*-like element was chosen for transient promoter deletion experiments, as the *pZCT1::GFP* expression in transgenic hairy roots was induced by JA and auxin."

379-381: This statement gets a bit confusing because the reader wonders whether high levels of *methyl jasmonate* correlate with a reduction of the substances mentioned (secologanin, strictosidine, and tabersonine), or whether strong induction of *ZCT1* correlates with a reduction of the important substances, or whether an experiment has been performed in the past that treats with high levels of MJ and then shows that ZCT1 expression strongly increases over a control state while at the same time the amount of the substances substantially reduces as compared to the control state. The reference Goklany et al, 2013 doesn't seem to mention ZCT1 in the main text. It would be helpful to clarify exactly what has been shown in the literature here.

Response: We have added to the following sentences to the introduction for further clarification (lines 49-60) and edited the sentences you are referring to in the discussion (lines 412-420):

In the introduction: "ZCTs repress the expression of at least two of the key MIA biosynthetic genes, strictosidine synthase (*STR*) and tryptophan decarboxylase (*TDC*), in transient expression assays (Pauw et al., 2004; Mortensen et al., 2019). ZCTs potentially limit the extent of MIA biosynthesis induced by jasmonate. For instance, optimum dosages of jasmonate (up to 250 μM; (Lee-Parsons, Ertürk and Tengtrakool, 2004; Goklany et al., 2013) enhance MIA biosynthesis and are correlated with a high ratio of transcriptional activators (*ORCAs*) to repressors (*ZCTs*) levels (Goklany et al., 2013). But higher dosages of jasmonate (> 500 μM; Lee-Parsons, Ertürk and Tengtrakool, 2004; Goklany et al., 2013) inhibit MIA biosynthesis and are correlated with a high ratio of transcriptional repressors (*ZCTs*) to activator (*ORCAs*) levels (Goklany et al., 2013). The inhibition of MIA biosynthesis with high jasmonate dosages is potentially mediated through repressors like ZCTs."

In the Discussion: "However, little has been shown regarding the hormonal regulation of *ZCT1* expression. We previously showed that *ZCT1* expression was strongly induced with high dosages of MJ (1 mM) in hairy root cultures (Goklany et al., 2013). Here, we showed that the -914 bp to +86 bp region upstream of *ZCT1* also leads to strong induction with auxin (1-NAA) (Figure 3), as well as with MJ in transgenic hairy roots."

Editor comments:

In addition to the comments by Reviewer 1, here are a couple of points that need to be addressed.

To establish a more complete analysis of the ZCT promoter sequences it would be useful to use PlantPAN (http://plantpan.itps.ncku.edu.tw/promoter.php).

Thank you for the advice. We have performed a PlantPAN 3.0 promoter analysis for the *ZCT1*, *ZCT2*, and *ZCT3* promoters. Results are added to the manuscript (lines 116-1120; 273-276, 336-337) and the supplementary sequence files (.gbk files). Citations are added for PlantPAN 3.0 and PlantCARE.

Since initially there seem to be some discrepancies between experiments in which only "hairy roots" are used and transient expression systems using whole seedlings it would be useful to mention the differences between A. rhizogenes transformation for the generation of hairy roots and A. tumefaciens GV3101 transformations when describing these experiments in the results section. This fact make it also difficult to differentiate between tissue specific effects and hormone signaling in these experiments. This point should be explained when speculating about potential tissue specific effects in the discussion section. Any information on the tissue specific expression of MIA biosynthesis genes would help provide additional context.

We agree that the different tissues (hairy roots versus seedlings) used in this study do not allow for an-apple-to-apple comparison. Therefore, we addressed the effect of a hormone only when the same tissue could be compared; for instance, we addressed the effect of auxin on *ZCT1* expression in seedlings by stating that *ZCT1* expression increased in seedlings transiently transformed with *A. rhizogenes* (which introduces auxin sensitivity genes) but not with disarmed *A. tumefaciens* (lines 436-443):

"Additionally, the responsiveness of the *ZCT1* promoter to auxins explains why *ZCT1* levels were increased during transient transformation of *C. roseus* seedlings with *A. rhizogenes* strain R1000 (Weaver et al., 2014), which transfers genes for auxin biosynthesis into plants (Inzé et al., 1984). *ZCT1* levels were not increased during transient transformation of *C. roseus* seedlings with the *A. tumefaciens* strain GV3101, which has been disarmed of its endogenous plant hormone biosynthetic genes (Figure 7)."

To discuss potential tissue specific or developmental specific role of the GARC on *ZCT1* expression, we discussed the context in which the GARC cluster bounded by W-boxes does have an effect (thanks for your suggestion) instead of comparing the contribution of this cluster in hairy roots versus seedlings (lines 456-461):

"However, GA_3 did not induce GFP expression in hairy roots (Figure 3); these potential discrepancies could be attributed to a condition-specific role of the GARC. For instance, in rice aleurone cells, the expression of the amylase gene is regulated by the GARC cluster bounded by

W-boxes, promoting the breakdown of starch in germinating seedlings in the presence of GA_3 (Xie et al., 2006)."

As the reviewer points out the effect of jasmonate on *ZCT* expression is unclear. It would be useful to clarify that in the introduction. The sentence in Line 46 " are expressed by the stress-induced...", is unclear. Please specify whether treatment by jasmonate leads to an induction or repression of these genes in the introduction. As the reviewer mentions, there is also some confusion on this point in the discussion section.

Response: We have added to the following sentences to the introduction for further clarification (lines 49-60) and edited the discussion (lines 413-420):

In the introduction: "*ZCTs* repress the expression of at least two of the key MIA biosynthetic genes, strictosidine synthase (*STR*) and tryptophan decarboxylase (*TDC*), in transient expression assays (Pauw et al., 2004; Mortensen et al., 2019). ZCTs potentially limit the extent of MIA biosynthesis induced by jasmonate. For instance, optimum dosages of jasmonate (up to 250 μM; (Lee-Parsons, Ertürk and Tengtrakool, 2004; Goklany et al., 2013) enhance MIA biosynthesis and are correlated with a high ratio of transcriptional activators (*ORCAs*) to repressors (*ZCTs*) levels (Goklany et al., 2013). But higher dosages of jasmonate (> 500 μM; Lee-Parsons, Ertürk and Tengtrakool, 2004; Goklany et al., 2013) inhibit MIA biosynthesis and are correlated with a high ratio of transcriptional repressors (*ZCTs*) to activator (*ORCAs*) levels (Goklany et al., 2013). The inhibition of MIA biosynthesis with high jasmonate dosages is potentially mediated through repressors like ZCTs."

In the Discussion: "However, little has been shown regarding the hormonal regulation of *ZCT1* expression. We previously showed that *ZCT1* expression was strongly induced with high dosages of MJ (1 mM) in hairy root cultures (Goklany et al., 2013). Here, we showed that the -914 bp to +86 bp region upstream of *ZCT1* also leads to strong induction with auxin (1-NAA) (Figure 3), as well as with MJ in transgenic hairy roots."

Line 54: A transition sentence mentioning that ZCT transcription factors belong to the C2H2-type zinc fingers would be useful for the non-expert reader.

Response: Added a sentence to emphasise that ZCTs belong to C2H2-type zinc fingers (line 65).

Figure 1 should be the first main figure cited. Just mention Figure S1 in line 257.

Response: We removed the reference to Figure 4 and are just mentioning Figure S1 (lines 273-274).

Figure 3: legend should include a description for the "untreated" sample.

Response: Added "No ethanol and no hormones were added to the untreated samples." (lines 744-745)

Statistical analyses: Results in Figure S2 require statistical analysis.

Response: Statistical test (Student's t test) was added to Figure S2.

Dunnet and Tukey-Kramer are posthoc test that are used after 1-way-ANOVA. This should be mentioned.

Response: That the data was analysed with a one-way ANOVA before performing the posthoc test was added to the Figures 3, 4, 5, 6, and 7.

Figures with RT-qPCR data should include in the legend a brief description of how "expression " was calculated, including details on the control gene and any other type of normalization.

Response: added the lines 812-815: "Transcript levels were normalized to the housekeeping gene, *SAND* (Pollier et al., 2014), and fold changes were calculated according to the 2-ΔΔCt method relative to the *GUS* control (Livak and Schmittgen, 2001)."

Line 310: to which figure does this description refer to?

Response: We added the Figure S3 (former Figure S3 is now referred to as Figure S4). This is the same data as for Figure 7, but instead of calculating the relative expression to a control condition, the relative abundance of *ORCA3* and *ZCT1* is calculated in comparison to the reference gene *SAND*.

Line 320: "For comparison to a series of deletion constructs..." This detail should be included earlier in the text, when first describing Figure 4. Although it is also sufficient in the figure legend.

Response: Deleted the sentence and left the description of the reference in Figure 4.

The effect of GA is rather small under the conditions use, it is strange to then mention in line 323 that the GA responsive complex is an "important regulatory" element. Are other conditions under which the GA response is stronger?

Response: We agree that the effect of GA and the GARC is small in the tested conditions. We removed therefore the according sentence (lines 355-357).

But there might be conditions under which the GA response might be stronger and we have added therefore the following sentences to the discussion (lines 456-461):

“However, GA_3 did not induce GFP expression in hairy roots (Figure 3); these potential discrepancies could be attributed to a condition-specific role of the GARC. For instance, in rice aleurone cells, the expression of the amylase gene is regulated by the GARC cluster bounded by W-boxes, promoting the breakdown of starch in germinating seedlings in the presence of GA_3 (Xie et al., 2006).”

Line 333: The text explains that "a portion of the GARC" is deleted in the pZCT1_243 construct. However, since the elements 1.GA, 2. GA, 3. GA in seems that the whole GARC is removed. Please clarify.

Response: Yes, the whole GRAC is deleted. We therefore removed “a portion of” from the sentence (line 367).

Line 352: The unpublished yeast 1-hybrid data should be shown or not mentioned.

Response: We deleted the sentence mentioning the yeast 1-hybrid data (lines 385-387).
